# Genetic relatedness of *Leptospira interrogans* serogroup Autumnalis isolated from humans, dogs, and mice in Japan

**DOI:** 10.1186/s13104-020-05211-1

**Published:** 2020-08-03

**Authors:** Nobuo Koizumi, Hidemasa Izumiya, Makoto Ohnishi

**Affiliations:** Department of Bacteriology I, National Institute of Infectious Diseases, 1-23-1 Toyama, Shinjuku, Tokyo, 162-8640 Japan

**Keywords:** Dog, *Leptospira interrogans*, Leptospirosis, Maintenance host, MLVA, Mouse

## Abstract

**Objective:**

Leptospirosis is a zoonotic disease caused by pathogenic spirochetes of *Leptospira* spp., and peridomiciliary rodents are the most important reservoir animals for human infection. Dogs are known to be the reservoir animal of *L. interrogans* serovar Canicola, but the importance of dogs in zoonotic transmission of other *Leptospira* serotypes/genotypes remains unclear. This study reports the isolation of *L. interrogans* serogroup Autumnalis from two human patients in Japan and describes the genetic comparison between canine and mouse isolates using multiple-locus variable-number tandem repeat analysis (MLVA).

**Results:**

MLVA revealed that 8 out of the 11 loci compared were identical between the two human isolates. The human isolates clustered with the dog but not the mouse isolates. Moreover, the profile of one of the human isolates was identical to that of one of the dog isolates.

## Introduction

Leptospirosis is a zoonotic disease caused by infection with the pathogenic spirochetes, *Leptospira* spp. [1, 2]. Human leptospirosis is an acute febrile illness with an extremely broad clinical spectrum ranging from influenza-like illness to severe disease forms characterized by jaundice, bleeding, renal failure, and death [1, 2]. Leptospires colonize the proximal renal tubules of maintenance hosts and are excreted in urine [2, 3]. Leptospirosis in humans is mainly contracted by exposure to water or soil contaminated with the urine of infected animals. Peridomiciliary rodents are important maintenance hosts for human infection [1, 2]. However, for *Leptospira interrogans* serovar Canicola, dogs serve as the primary maintenance host and pose potential zoonotic transmission to humans [4].

Historically, clinical *Leptospira* isolates were characterized via serology. There are more than 300 serovars in the genus *Leptospira,* and antigenically related serovars are classified into serogroups [1]. However, serovar identification is currently extremely difficult, therefore most clinical isolates are characterized at the serogroup level. Therefore, molecular typing has become the main method for the characterization of *Leptospira* isolates [5]. Multi-locus sequence typing is a highly reliable and reproducible method for the molecular typing of pathogenic *Leptospira* species [6]. Multiple-locus variable-number tandem repeat analysis (MLVA) has an excellent discrimination power in *L. interrogans* and its results are concordant with those from serotyping [7, 8]. In our previous studies, we isolated *L. interrogans* serogroup Autumnalis from large Japanese field mice (*Apodemus speciosus*) and dogs in Miyazaki, Kagoshima, Saga, Hokkaido, Aomori, Akita, Fukushima, and Nagano Prefectures, Japan [9, 10]. MLVA performed on the mouse and dog isolates demonstrated that they are of different genotypes (MLVA type) [7, 8].

In the present study, we isolated *L. interrogans* serogroup Autumnalis from two human patients and conducted a genetic comparison between canine and mouse isolates using MLVA.

## Main text

Leptospires were isolated from two human patients using blood culture in liquid Korthof’s medium in 2011 (case 1) and 2013 (case 2). The patient in case 1 was a female in her 60 s. She engaged in rice-harvesting in Miyazaki Prefecture 3 weeks before the onset of the disease. At the time of hospitalization, she presented with fever (40.1 °C), vomiting, diarrhea, conjunctival suffusion, and jaundice. Her laboratory tests revealed thrombocytopenia (7.0 × 10^4^/μL) and elevated total bilirubin (1.3 mg/dL) and serum creatinine (0.9 mg/dL) levels. Piperacillin sodium was administered to the patient for treatment. Blood was inoculated into liquid Korthof’s medium 2 days after the disease onset and *Leptospira* sp. was isolated 20 days after the inoculation. The patient in case 2 was a schoolboy in his 10 s. He had undertaken a recreational activity in a river in Kagoshima Prefecture adjoining west of Miyazaki Prefecture 10 days prior to the disease onset. He presented with fever (39.9 °C), headache, and myalgia at the time of hospitalization. His laboratory tests revealed proteinuria and thrombocytopenia (9.5 × 10^4^/μL). Ceftriaxone was administered. Heparinized blood collected 3 days after the disease onset was transferred to our institute, where the blood was inoculated into liquid Korthof’s medium. *Leptospira* sp. was isolated 17 days after the inoculation.

**Fig. 1:**
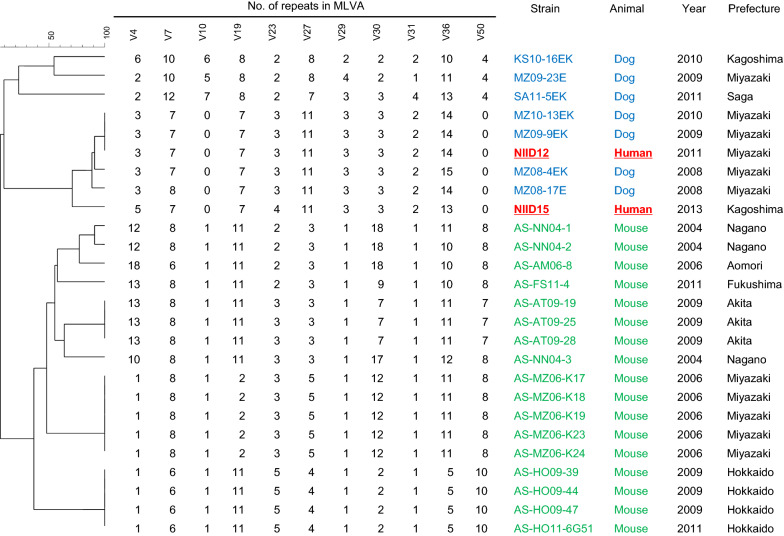
A dendrogram based on multiple-locus variable-number tandem repeat analysis (MLVA) using 11 loci showing relationships among human, dog, and mouse isolates of *L. interrogans* serogroup Autumnalis. Profiles of the copy numbers of the repeats of the 11 loci were analyzed using a categorical multi-state coefficient and unweighted pair group method with arithmetic averages (UPGMA) as a clustering algorithm with BioNumerics software version 7.6 (Applied Maths, Belgium)

Leptospiral DNA was extracted using DNeasy Blood & Tissue Kits (Qiagen, Hilden, Germany) and subjected to PCR targeting the flagellar gene *flaB* of pathogenic leptospires, followed by DNA sequencing for species identification [9]. The *flaB* sequences of the isolates, NIID12 for case 1 and NIID15 for case 2, were identical to each other and the isolates were identified as *L. interrogans* (DDBJ accession numbers: LC521310 for NIID12 and LC521311 for NIID15). The serogroup of both isolates was identified as Autumnalis using the microscopic agglutination test and a panel of 18 antisera [9]. To investigate the genetic relatedness among human, murine, and canine isolates of *L. interrogans* serogroup Autumnalis, MLVA was performed for 11 loci [7] on the two human isolates, and their profiles were compared with those of the murine and canine isolates. The sizes of the amplicons were converted to repeat copy numbers for analysis using a categorical multi-state coefficient and unweighted pair group method with arithmetic averages (UPGMA) as a clustering algorithm with BioNumerics software version 7.6 (Applied Maths, Belgium). MLVA showed that 8 out of the 11 loci were identical between the two isolates. Interestingly, the human isolates clustered with the dog but not mouse isolates. Moreover, the profile of NIID12 was identical to that of one of the dog isolates (Fig. 1). The genetic diversity among *Leptospira* serogroups is variable, and the host animal is suggested to be an important factor in *Leptospira* diversification [8, 11]. Genetic diversity in *L. interrogans* serogroup Autumnalis was observed among canine isolates, which could explain the genetic difference observed between the human isolates. The medical research ethics committee of the National Institute of Infectious Diseases for the use of human subjects exempts their reviews for the characterization of leptospiral isolates obtained by laboratory diagnosis when requested formally by prefectural governments.

This study revealed that human isolates are genetically identical or similar to canine but not mouse isolates (Fig. 1). Although peridomiciliary rodents are the most important reservoir animals for human infection and we had thought they were the most possible infection source of *L. interrogans* serogroup Autumnalis for humans in Japan, this study demonstrated that other animals than mouse may constitute the reservoir animal for this genotype of *L. interrogans* serogroup Autumnalis. *L. interrogans* has been isolated from brown rats and raccoons in Japan, but this genotype strain has never been isolated from these animals [8, 12]. *L. interrogans flaB* sequences have been detected in wild boars throughout Japan [13]. One of the *flaB* sequences (691 bp) detected in wild boar was identical to those of *L. interrogans* serogroup Autumnalis human isolates in this study, although that particular *flaB* sequence has been detected in different serogroups of *L. interrogans*.

Dogs are known to be the reservoir animal of *L. interrogans* serovar Canicola [4]. There are several reports of possible dog-to-human transmission of *L. interrogans* serovar Canicola, indicating the important role of dogs in zoonotic transmission of leptospirosis [14–18]. However, the widespread use of bivalent vaccines containing serovar Canicola is likely to decrease the circulation of serovar Canicola among dogs in the U.S., Europe, and Japan [7, 19, 20]. Other *Leptospira* species and serovars have been detected in asymptomatic dogs, but it remains unclear whether these dogs serve as the maintenance host of other genotypes/serotypes and facilitate zoonotic transmission to humans [14, 15]. It is possible that both humans and dogs are incidental hosts, and other animals, such as wild boars, maintain this genotype of *L. interrogans* serogroup Autumnalis. However, the identical or similar MLVA profile in human and canine isolates may suggest that dogs are the reservoir animal of this genotype of *L. interrogans* serogroup Autumnalis and a source for human infection. The canine isolates described in this study were obtained from symptomatic dogs [9]. *L. interrogans* serovar Canicola causes acute and subacute hepatic and renal failure in dogs, and the dogs become asymptomatic carriers of this serovar strain after recovering from acute infection [21]. Although we have not investigated the carrier status of this genotype of *L. interrogans* serogroup Autumnalis among asymptomatic dogs, as with serovar Canicola infection, infected dogs would be chronic carriers of this genotype strain after recovery.

## Limitations

The study was conducted on only two human isolates. We did not investigate the carrier status of this genotype of *L. interrogans* serogroup Autumnalis among asymptomatic dogs. We did not obtain *L. interrogans* isolate from wild boars and its serological and detailed genetic characteristics remain unknown. Therefore, we cannot conclude on which animal(s) is the reservoir animal of this genotype of *L. interrogans* serogroup Autumnalis.

## Data Availability

The *flaB* sequences have been deposited in a public database (DDBJ accession numbers LC521310 and LC521311).

## Abbreviations

MLVA: Multiple-locus variable-number tandem repeat analysis
UPGMA: Unweighted pair group method using arithmetic averages
